# Validation and Testing of Fast Healthcare Interoperability Resources Standards Compliance: Data Analysis

**DOI:** 10.2196/10870

**Published:** 2018-10-23

**Authors:** Jason Walonoski, Robert Scanlon, Conor Dowling, Mario Hyland, Richard Ettema, Steven Posnack

**Affiliations:** 1 The MITRE Corporation Bedford, MA United States; 2 AEGIS.net, Inc Rockville, MD United States; 3 The Office of the National Coordinator for Health Information Technology US Department of Health and Human Services Washington, DC United States

**Keywords:** electronic health records, health data interoperability, test-driven development, practice makes perfect

## Abstract

**Background:**

There is wide recognition that the lack of health data interoperability has significant impacts. Traditionally, health data standards are complex and test-driven methods played important roles in achieving interoperability. The Health Level Seven International (HL7) standard Fast Healthcare Interoperability Resources (FHIR) may be a technical solution that aligns with policy, but systems need to be validated and tested.

**Objective:**

Our objective is to explore the question of whether or not the regular use of validation and testing tools improves server compliance with the HL7 FHIR specification.

**Methods:**

We used two independent validation and testing tools, Crucible and Touchstone, and analyzed the usage and result data to determine their impact on server compliance with the HL7 FHIR specification.

**Results:**

The use of validation and testing tools such as Crucible and Touchstone are strongly correlated with increased compliance and “practice makes perfect.” Frequent and thorough testing has clear implications for health data interoperability. Additional data analysis reveals trends over time with respect to vendors, use cases, and FHIR versions.

**Conclusions:**

Validation and testing tools can aid in the transition to an interoperable health care infrastructure. Developers that use testing and validation tools tend to produce more compliant FHIR implementations. When it comes to health data interoperability, “practice makes perfect.”

## Introduction

### Lack of Health Interoperability

Despite the relatively rapid nationwide adoption of electronic health records (EHRs), the industry’s ability to successfully exchange computable health data has not kept pace. A recent study found that less than 35% of providers report data exchange with other providers within the same organization or affiliated hospitals. The exchange of data across organizations is even more limited, with less than 14% of providers reporting they exchange data with providers in other organizations or unaffiliated hospitals [1]. Limited health data interoperability has significant impacts. As part of providing care for their patients, the typical primary care physician (PCP) coordinates care with 229 other physicians across 117 organizations [2]. Limited interoperability makes the already complicated problem of care coordination even more challenging. Currently, 40% of PCPs report that when they refer a patient to a specialist, they do not efficiently receive the outcomes of the visit, including cases where the patient's plan of care or active medications have changed [3]. The lack of interoperability leads to gaps in critical information at the point of care. This puts undue burden on patients who currently must fill those gaps in data and when those gaps remain unfilled, they can lead to significant safety issues. An inpatient study found that 18% of medical errors leading to adverse drug events could be traced back to missing data in the patient's medical record [4]. Finally, limited health care data interoperability has an immense cost. For example, West Health Institute, an independent, nonprofit medical research organization, estimates that the lack of medical device interoperability alone leads to over US $30 billion in wasteful spending each year. Beyond medical devices, the broader problem of limited health care data interoperability further contributes to an estimated US $700 billion in wasteful spending annually in health care [5].

### Improving Interoperability

As indicated by the JASON report [6], the implications and benefits from a truly open digital health care architecture are wide ranging, from enabling individual patients to obtain, share, and authorize who can view their data, to population health analytics and research. Currently, data and exchange standards in health care do not adequately ensure out-of-the-box interoperability, chiefly due to the complexity and lack of identical interpretations of the published standards by health IT software developers. Rigorous testing and validation will help move the US health care system in the direction of open, accessible, patient-centric care.

To date, the health care community has produced and tolerated data standards that are complex, difficult to understand, and technically challenging to consistently implement and test. While well meaning, such standardization efforts have advanced interoperability only so far and, at the same time, stifled innovation due to high custom development and maintenance costs. In one such situation, MITRE has previously demonstrated in the domain of clinical quality measurement that a test-driven approach can successfully establish a framework for interoperability using national health care standards [7]. Similarly, AEGIS.net has successfully supported health information networks that focus on nationwide scale and standards adoption with its Developers Integration Lab cloud-based Test-Driven-Development (TDD) Test Platform [8].

### Health Level Seven International Fast Healthcare Interoperability Resources

The Health Level Seven International (HL7) Fast Healthcare Interoperability Resources (FHIR) standard [9] offers a better and more innovation-friendly path forward. The FHIR standard is designed to be easily and quickly implemented while also, over time, supporting a broad set of health care use cases. It is also a rapidly evolving standard that consists of data formats for health care resources and an application programming interface (API) for the exchange of this information between client applications and servers. This API uses Representational State Transfer web services, a modern technology pattern that powers most of today’s Internet.

Although the Office of the National Coordinator for Health Information Technology’s (ONC) 2015 Edition Health IT Certification Criteria includes an API certification criterion—45 United States Code of Federal Regulations 170.315(g)(8) and (g)(9)—that is well suited for FHIR implementation, no government regulations require health IT developers to conform to any published version of the FHIR standard [10]. To facilitate this without regulation, a group of commercial health IT developers established the Argonaut Project. Argonauts have committed to promote health interoperability by using FHIR within the industry by defining implementation guides and profiles for read-only data access and document retrieval [11].

### Policy Context

In 2015, the ONC published the document, Connecting Health and Care for the Nation: A Shared Nationwide Interoperability Roadmap (the Roadmap) [12]. At a high level, the Roadmap expressed that interoperability needed to focus on three overall themes: (1) a supportive payment and regulatory environment, (2) policy and technical components, and (3) outcomes that could be measured and could impact individuals and providers. Section G of the Roadmap laid out the need for an industry-wide testing and certification infrastructure, stating that a “diverse and complementary set of testing and certification programs will need to be in place to achieve nationwide interoperability.” Further, with respect to testing, the Roadmap indicated that “the health IT ecosystem will need to invest in more efficient ways to test health IT that is implemented and used among a diverse set of stakeholders.”

The 21st Century Cures Act (the Cures Act), Public Law 114-255 [13], was signed into law in late 2016. The Cures Act includes policies that impact everything from medical devices to precision medicine. In the context of health IT, it includes the most substantial update to the ONC’s authority since the Health Information Technology for Economic and Clinical Health Act was passed in 2009. Specifically, the Cures Act includes a statutory definition for *interoperability*; it establishes a new federal advisory committee, the Health IT Advisory Committee, it requires the National Coordinator to amend the ONC Health IT Certification Program to adopt *conditions of certification* that are applicable to health IT developers, and it defines *information blocking* and the penalties associated with doing so.

Importantly, and relevant to this paper, the Cures Act includes two provisions within the conditions of certification related to APIs. First, it charges ONC to require that health IT developers publish APIs that can enable health information to be accessed, exchanged, and used “without special effort.” Second, it charges ONC with requiring that health IT developers successfully test the real-world use of their certified technology for interoperability in the type of setting in which the technology is marketed. Taken together, these two statutory requirements signal a growing need for the industry to coalesce and invest in API-testing capacity.

### Objectives

To meet the requirements expressed within the Cures Act, health IT developers need substantive tools to validate and test system conformity to the FHIR specification. Furthermore, the consistent implementation of FHIR will help enable an open and innovation-friendly ecosystem that can make data exchange more efficient and reduce interface costs. Both Crucible and Touchstone projects represent production-ready testing platforms for the FHIR specification, which can immediately be leveraged by industry to support their needs for FHIR-based testing [14,15].

Other available testing tools include Sprinkler, an open-source project developed by Firely, that tested FHIR servers with a web-based application [16]. Sprinkler has been retired and has not been publicly updated in four years. Another FHIR test tool available to the community is the Sync for Science Test Suite, which provides similar FHIR testing capabilities, but is geared specifically for the Sync for Science program [17].

The objective of this research was to examine whether or not the use of validation and test tools, specifically Crucible and Touchstone, had any impact on vendor compliance with the FHIR specification and, by extension, interoperability.

## Methods

### Overview

Two independent projects—MITRE’s Crucible project and AEGIS.net’s Touchstone project—provide the capability to rigorously test servers against the FHIR specification. Such testing assures health IT developers and app developers that the standards have been consistently implemented and deployed. This kind of testing is essential to enable interoperable health IT solutions that can be used to deliver safer and more efficient health care.

### MITRE Crucible Project

Crucible is a set of open-source testing tools for HL7 International FHIR developed by MITRE through an internally funded research program. It is provided as a free and public service to the FHIR development community to promote correct FHIR implementations. Its capabilities include the testing of servers for conformance to the FHIR standard, scoring patient records for completeness, and generating synthetic patient data suitable for testing [14].

The Crucible tool has been used by FHIR developers in the health care information technology industry since 2015. Developers can test their FHIR implementations through the Crucible website [14] by entering a URL at which their server can be accessed via the FHIR API.

There are three ways that Crucible can be used to test server compliance:

1. Server compliance tests may be manually run through the public instance of Crucible.
2. Server compliance tests of known servers are automated to run every 3 days through the public instance of Crucible.
3. Server compliance tests may be run on private instances of Crucible behind a private firewall.

In our analysis, this paper examines test results from manual and automated tests run through the public instance. Only manually run tests are considered as an indicator of system usage. Tests run on private instances of Crucible are not included, as that data is not available to the researchers.

### AEGIS.net, Inc Touchstone Project

Touchstone is an open-access platform which combines nearly 20 years of automated lab-based testing initiatives, most recently the cloud-based Test-as-a-Service Developers Integration Lab developed by AEGIS.net through internal research and development. By leveraging the experience gained and lessons learned supporting ONC onboarding participant organizations to the early stages of Nationwide Health Information Network and later hosting the Sequoia Project formal testing program for eHealth Exchange, AEGIS.net has advanced this test platform to address FHIR [15].

Touchstone has successfully been used by developers and quality assurance experts in health care information technology since 2015. Users can privately test their FHIR implementations by navigating to the Touchstone Project site [15] and create an account to support testing FHIR Implementations. Publication of any Touchstone test results only occurs with a developer’s prior approval.

In order to test for conformance and interoperability, Touchstone combines the following features in an open-access platform:

1. Testing both client applications and server implementations, while supporting peer-to-peer, multi-actor scenarios (ie, care coordination and workflow) in a unified testing approach.
2. Testing is based entirely on the FHIR Test Script Resource, allowing for crowdsourcing future test case development.
3. Multi-version FHIR support, which facilitates testing backwards compatibility and future-proofing systems and products to ensure a continuously interoperable ecosystem.

To gauge FHIR implementation conformance, this paper examines test results from manual and API-automated tests run through the public cloud instance of Touchstone. Only vendor-initiated tests against the cloud version of Touchstone are considered as an indicator of system usage. Tests run on private instances of Touchstone are not included as that data is not available to the researchers.

### Testing Period

The FHIR specification was originally proposed as a new health care data and exchange standard in August 2011. The first official release as a Draft Standard for Trial Use (DSTU) was published on September 30, 2014. Subsequent official releases of the FHIR specification have occurred on a 1.5-2-year balloting cycle. The FHIR specification has rapidly evolved over a short number of years; until initial stabilization of the specification occurred with the release of the DSTU, the introduction of publicly available testing tools was not feasible. To that point, the Crucible and Touchstone platforms only became available starting in 2015 when the test execution results data used in this statistical analysis began to be collected. For this study, data from Crucible ranged from December 1, 2015, to May 31, 2017, and data from Touchstone ranged from September 27, 2015, to September 3, 2017.

### Data Collection

Data was collected for this study through the usage of the Crucible and Touchstone projects. During the study period, software developers executed tests using both projects either autonomously or as part of a FHIR Connectathon. Both projects automatically collected usage data on the tests that they execute. This included the following: which FHIR server was under test, the version of FHIR being tested, which tests were being executed and how those tests map to the FHIR specification, the results of each test (eg, pass, fail, skip), as well as step-by-step interactions between the testing system and the target FHIR server (eg, every HTTP request including headers and body and every HTTP response including headers and body), and detailed introspection and checks of those results.

## Results

We wanted to know whether or not there was a relationship between testing and compliance. Therefore, we explored whether a statistically significant correlation could be found between the frequency with which vendors execute tests and their conformance with the FHIR specification. For this regression, servers were grouped together by vendor and as many vendors tested FHIR implementations using multiple servers. The number of manual tests executed was used as a measure of an organization’s usage level. The number of distinct test suites supported (ie, tests successfully passed) across all the servers was used to measure vendor performance. This metric is a good approximation of the number of features a vendor has implemented successfully and completely.

The number of tests executed were log-normalized to reflect decreasing marginal returns. This is because the most complex test suites tend to be implemented by developers last and require more implementation hours and testing. Regressing log tests executed against the number of supported suites gives a statistically significant (*P*<.005, n=115) positive correlation between Crucible usage and vendor performance. In other words, using linear regression to predict vendor performance (ie, number of test suites passed), it was found that the number of tests executed (beta=.80, *P*<.005) was a significant predictor. The model fit was R-squared=.262.

A similar analysis for Touchstone shows a statistically significant (*P*<.005, n=70) positive correlation between Touchstone usage and vendor performance. In other words, using linear regression to predict vendor performance (ie, number of unique tests passed), it was found that the number of tests executed (beta=0.11, *P*<.005) were significant predictors. The model fit was R-squared=.883.

**Figure 1:**
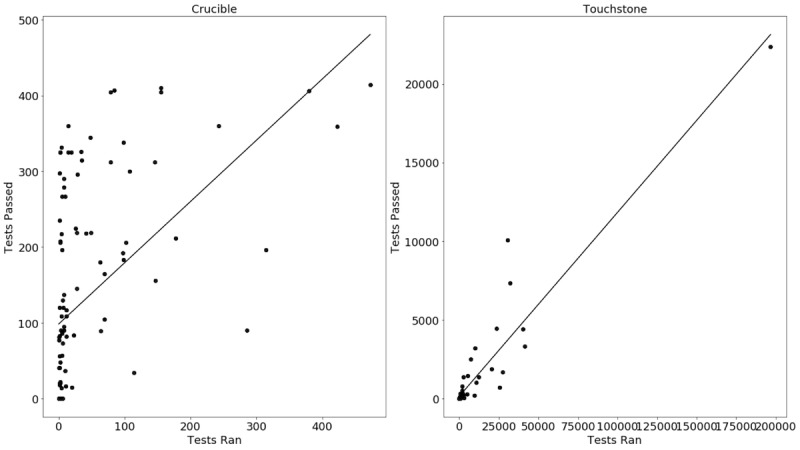
Predicting suites passed by tests executed.

**Figure 2:**
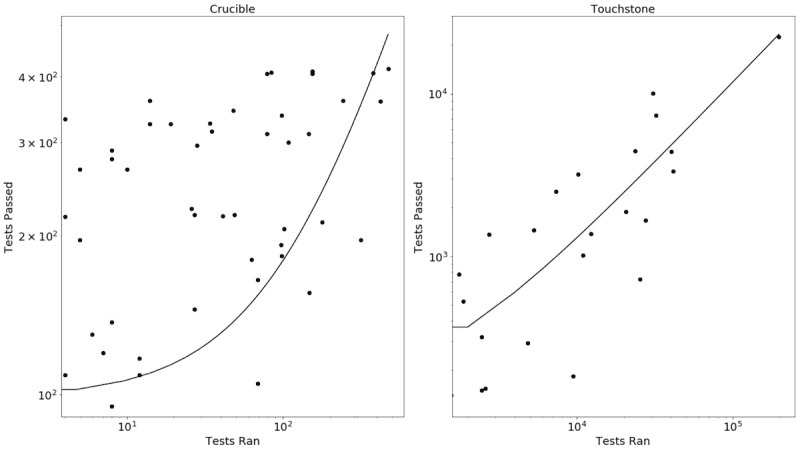
Predicting suites passed by tests executed (log scale).

These simple regressions—plotted in Figure 1 in a linear scale and Figure 2 with a log scale—indicate that committed FHIR developers are gaining value from Crucible and Touchstone through repeated use of testing services and incremental improvements of their implementations. In other words, the classic adage “practice makes perfect” unsurprisingly proves true. Test-driven development (*practice*) leads to improved specification adherence (*perfection*).

## Discussion

### Principal Findings

The results of our data analysis indicate that as the frequency of testing or number of tests increases, the performance of a server against those tests increases. This should not be surprising as software developers will address issues and fix defects in order to pass the tests, so long as they are discovering these issues and defects by repeated testing. Assuming the tests accurately and adequately cover the depth and breadth of the FHIR specification, then FHIR servers developed and tested using these tests in a test-driven manner should more accurately adhere to the FHIR specification. If compatibility with FHIR equates to health data interoperability, then it seems that fair and neutral testing is critical to achieving that goal. Of course, health data interoperability is vastly more complex than FHIR alone; other factors include, but are not limited to, clinical terminologies, security and trust frameworks, clinical workflow compatibility, and financial incentives. But the correct implementation of software that adheres to the FHIR specification is a good first step to exchanging data.

### Tests Over Time

**Figure 3:**
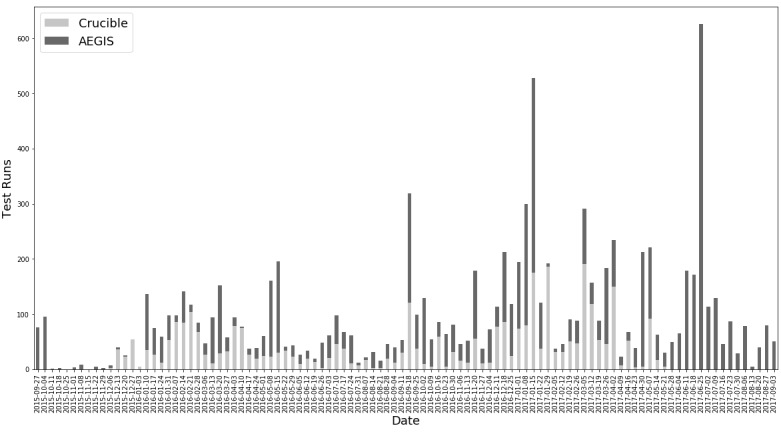
Test runs per week.

As shown in Figure 3, Crucible has seen use since its launch, with a period of high usage during February 2016, corresponding with increased Argonaut testing and spikes in usage during HL7 Connectathons. Over its lifetime, Crucible has averaged just over 42 test executions per week with testing volume trending upward over time. Touchstone has also seen significant growth in use since its inception as FHIR has grown in popularity and importance.

### Tracking Vendors Over Time

**Figure 4:**
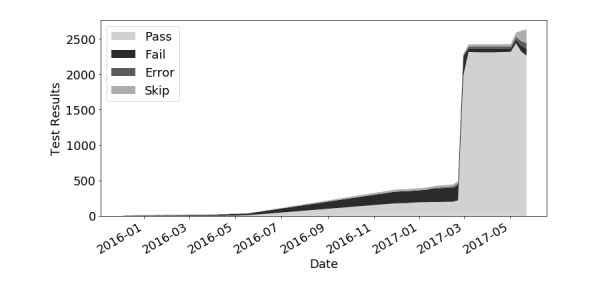
Vendor A STU3 (Standard for Trial Use version 3) servers.

Using regular automated testing on known FHIR servers, Crucible can track the progress of a server over time. Because vendors often use temporary server URLs for testing purposes, to track the weekly progress of an individual vendor, we can aggregate the results of all known servers for that vendor and use the best results to track their progress implementing FHIR. Crucible’s tracking of one anonymized vendor’s Standard for Trial Use version 3 (STU3) servers is shown below in Figure 4. They show a slow improvement before a drastic increase in performance in February 2017.

Similarly, looking specifically at the top active anonymized users of Touchstone—Vendor A (188 uses), who started testing with Touchstone in February 2017, Vendor B (378 uses), Vendor C (321 uses), and Vendor D (207 uses)—there is evidence of both high use of Touchstone and improvement in their FHIR implementations. These implementations used Touchstone consistently during the study period, with their results progressively improving (ie, passing less than 20 tests initially to passing over 1000 tests). Touchstone’s TDD testing capabilities allowed these developers to implement their FHIR servers faster by finding errors and confirming the correctness of their implementations, including managing version upgrades.

It is important to note that Vendor A accomplished in 6 weeks what many organizations accomplish in 12-24 weeks, by leveraging TDD—and testing on a daily basis—and integrating continuous testing into their development lifecycle.

### Tests by Use Case

During the study period there were 3253 identified user-initiated test executions on Crucible, of which 1970 included only a single test suite. Four of the top 10 test suites executed were Argonaut suites. The other commonly executed suites include the most general tests: reading, searching, history retrieval, and formatting, as well as the transaction and batch test. The FHIR patient-resource test was the most-used resource test since it is one of the most important and central resources in the FHIR specification.

Within Touchstone, there were 529,847 tests run during the study period. A total of 99,848 (18.8%) of the tests executed were specifically testing the FHIR Patient Resource, while 55,163 (10.4%) tested the terminology functionality. Touchstone also includes tests for HL7 Connectathon tracks, which comprised 125,720 (23.7%) of the tests run by volume, although many of these tests were most likely run outside of Connectathons.

The top tests executed on Crucible and Touchstone are listed in Table 1.

### Tests by Version

FHIR is an evolving standard that has seen three major releases in the last 4 years and a dozen minor releases in the same time frame [9]. The topic of FHIR versioning has similarly evolved as various vendors begin to build and deploy production services. Only recently, after the study period, did HL7 add versioning information to the FHIR specification, at a maturity level of *not applicable* (N/A) and status of *informative*, meaning it is merely information and not rules to be followed [18,19]. Currently, and during the study period, both Touchstone and Crucible examined the FHIR Server Capability Statement to determine the declared version of FHIR supported.

**Figure 5:**
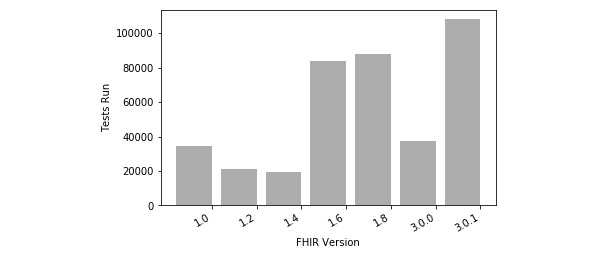
Touchstone usage by Fast Healthcare Interoperability Resources (FHIR) version.

**Figure 6:**
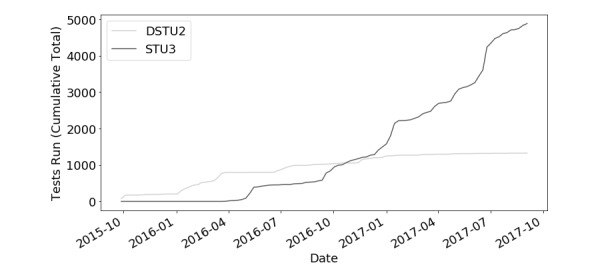
Touchstone Fast Healthcare Interoperability Resources (FHIR) by version over time. DSTU2: Draft Standard for Trial Use version 2; STU3: Standard for Trial Use version 3.

Crucible supports testing the last two major versions of FHIR, while Touchstone supports testing all point releases since FHIR 1.0. Figures 5 and 6 show the community shift from testing one version of FHIR to the next. Examining tests tagged with specific FHIR versions, we can see growth in testing usage throughout the lifetime of Touchstone. The dip in testing volume of FHIR 3.0.0 is indicative of the swift transition to FHIR 3.0.1.

### Community Engagement

Beyond providing the tools themselves, the Crucible and Touchstone teams have maintained considerable involvement with the FHIR development community by attending Connectathons sponsored by HL7, assisting the Argonaut group by providing tailor-made tests for their use cases, and in the case of Crucible, reaching out to the open-source community for involvement in the development of the software.

### Connectathons

The Crucible development team has attended each HL7-sponsored FHIR Connectathon since Connectathon 8 in January 2015 through Connectathon 17 in January 2018. The AEGIS.net team has attended each HL7-sponsored FHIR Connectathon since Connectathon 4 in September 2013 and introduced Touchstone at Connectathon 10 in October 2015. Both Crucible and Touchstone develop and support a suite of tests for each Connectathon, specific to that event’s tracks. The Touchstone team regularly runs a “Developers Introduction to FHIR” session parallel to each Connectathon introducing FHIR and TDD.

**Table 1:** Top tests executed by users.

Rank	Crucible		Touchstone	
	Test ID	Number of executions	Test ID	Number of executions
1	Argonaut Sprint 1	858	Patient Resource Test	132,328
2	Read Test	664	ValueSet Resource Test	59,162
3	Argonaut Sprint 3	549	Practitioner Resource Test	26,282
4	Argonaut Sprint 4	539	Organization Resource Test	22,124
5	History001	476	Location Resource Test	12,243
6	Search001	475	Observation Resource Test	11,592
7	Format001	460	Device Resource Test	10,698
8	Argonaut Sprint 5	451	AllergyIntolerance Resource Test	10,621
9	Transaction and Batch Test	447	Appointment Resource Test	10,232
10	Patient Resource Test	445	Condition Resource Test	9588

**Table 2:** Anonymized performance of Argonaut members at the completion of each Argonaut Sprint.

Vendor	Sprint			Resprint			Sprint				Argonaut Connectathon tests
	1	2	3	1	2	3	4	5	6	7	
E	Pass	Pass	Pass	Pass	Pass	Pass	Pass	Pass	Pass	Pass	Pass
F	Pass	Pass	Pass	Pass	Pass	Pass	Pass	Pass	Pass	Pass	Pass
G	Pass	Fail	Pass	Pass	Pass	Pass	Pass	Pass	Pass	Pass	Fail
I	Pass	Pass	Pass	Pass	Pass	Pass	Pass	Pass	Pass	Pass	Fail
M	Pass	Pass	Pass	Pass	Pass	Pass	Pass	Pass	Pass	Pass	Pass
N	Pass	Fail	Pass	Fail	Fail	Fail	Pass	Pass	Pass	Pass	Pass
O	Pass	Fail	Pass	Pass	Pass	Pass	Pass	Pass	Pass	Pass	Fail
P	Pass	Fail	Pass	Pass	Pass	Pass	Pass	Pass	Pass	Pass	Fail
Q	Pass	Pass	Pass	Pass	Pass	Pass	Pass	Pass	Pass	Pass	Pass
R	Pass	Pass	Pass	Pass	Pass	Pass	Pass	Pass	Pass	Pass	Pass
S	Fail	Fail	Fail	Fail	Fail	Fail	Fail	Fail	Fail	Fail	Fail
T	Pass	Pass	Pass	Pass	Pass	Pass	Pass	Pass	Pass	Pass	Pass
U	Pass	Pass	Pass	Pass	Pass	Pass	Pass	Pass	Pass	Pass	Pass
V	Fail	Fail	Fail	Fail	Fail	Fail	Fail	Fail	Fail	Fail	Fail

### Argonaut

The Argonaut Project is a private sector initiative with the mission of advancing industry adoption of modern open interoperability standards. Its stated purpose is to “develop a first-generation FHIR-based API and Core Data Services specification to enable expanded information sharing for electronic health records and other health information technology using existing Internet standards and architectural patterns and styles” [11].

With Touchstone’s and Crucible’s missions to advance the adoption of the FHIR API, both teams collaborated with Argonaut vendors to develop a series of test suites to help them test their FHIR implementations. Crucible’s test suite results show almost all Argonaut members failed these test suites initially. However, as shown in Table 2, many of the members now support most of the test suites. This could be attributed to the high volume of testing that was performed on the Argonaut suites during the initial Argonauts implementation sprints.

### Caveats

Electronic health records have structured and unstructured data. FHIR supports both of these data types: structured data using Resources and unstructured data using Binary and DocumentReference [20,21]. Neither Crucible nor Touchstone test for clinical correctness; they focus purely on technical correctness. Achieving health data interoperability may also require a higher level of interoperability (eg, semantic) beyond technical exchange.

### Conclusions

Crucible and Touchstone have proven to be valuable tools for the FHIR developer community. These tools can aid in the transition to an interoperable health care infrastructure by providing open reference implementations for FHIR testing and support future Cures Act requirements. Our research shows that developers that use testing and validation tools tend to produce more compliant FHIR implementations. The test data collected by MITRE and AEGIS.net during the study period shows that when it comes to health data interoperability, “practice makes perfect.” This gives us hope that a future with ubiquitous health care information interoperability is possible.

## Abbreviations

API: application programming interface
DSTU: Draft Standard for Trial Use
EHR: electronic health record
FHIR: Fast Healthcare Interoperability Resources
HL7: Health Level Seven International
ONC: Office of the National Coordinator for Health Information Technology
PCP: primary care physician
STU3: Standard for Trial Use version 3
TDD: Test-Driven-Development

## References

[1] Furukawa M, King J, Patel V, Hsiao C, Adler-Milstein J, Jha A (2014). Health Affairs.

[2] Pham H, O'Malley AS, Bach P, Saiontz-Martinez C, Schrag D (2009). Primary care physicians' links to other physicians through Medicare patients: The scope of care coordination. Ann Intern Med.

[3] Davis K, Stremikis K, Schoen C, Squires D (2014). Mirror, Mirror on the Wall: How the US Health Care System Compares Internationally. 2014 Update.

[4] Middleton B (2004). ResearchGate.

[5] (2013). The Value of Medical Device Interoperability: Improving Patient Care With More Than $30 Billion in Annual Health Care Savings.

[6] JASON, The MITRE Corporation (2014). A Robust Health Data Infrastructure.

[7] HealthIT.gov.

[8] AEGIS.

[9] Health Level Seven International.

[10] (2015). Federal Register: The Daily Journal of the United States Government.

[11] Health Level Seven International.

[12] (2015). Connecting Health and Care for the Nation: A Shared Nationwide Interoperability Roadmap.

[13] United States 114th Congress (2016). The 21st Century Cures Act, Public Law 114-255.

[14] Project Crucible.

[15] Touchstone.

[16] Sprinkler.

[17] S4S Test Suite.

[18] Health Level Seven International.

[19] Health Level Seven International.

[20] Health Level Seven International.

[21] Health Level Seven International.

